# Cancer-generated lactic acid: a regulatory, immunosuppressive metabolite?

**DOI:** 10.1002/path.4218

**Published:** 2013-07-09

**Authors:** Stephen Yiu Chuen Choi, Colin C Collins, Peter W Gout, Yuzhuo Wang

**Affiliations:** 1 Department of Experimental Therapeutics, BC Cancer Agency, VancouverBritish Columbia, Canada; 2 Department of Urologic Sciences, Faculty of Medicine, University of British ColumbiaVancouver, British Columbia, Canada; 3 Vancouver Prostate CentreVancouver, British Columbia, Canada

**Keywords:** Warburg effect, aerobic glycolysis, glutaminolysis, lactic acid, immune suppression, tumour micro-environment

## Abstract

The common preference of cancers for lactic acid-generating metabolic energy pathways has led to proposals that their reprogrammed metabolism confers growth advantages such as decreased susceptibility to hypoxic stress. Recent observations, however, suggest that it generates a novel way for cancer survival. There is increasing evidence that cancers can escape immune destruction by suppressing the anti-cancer immune response through maintaining a relatively low pH in their micro-environment. Tumours achieve this by regulating lactic acid secretion via modification of glucose/glutamine metabolisms. We propose that the maintenance by cancers of a relatively low pH in their micro-environment, via regulation of their lactic acid secretion through selective modification of their energy metabolism, is another major mechanism by which cancers can suppress the anti-cancer immune response. Cancer-generated lactic acid could thus be viewed as a critical, immunosuppressive metabolite in the tumour micro-environment rather than a ‘waste product’. This paradigm shift can have major impact on therapeutic strategy development. Copyright © 2013 Pathological Society of Great Britain and Ireland. Published by John Wiley & Sons, Ltd.

## Introduction

Deregulated proliferation of cancer cells is generally associated with altered energy metabolism. Glucose is a primary source of energy. Under aerobic conditions, normal cells metabolize glucose to pyruvate via glycolysis in the cytosol, and subsequently convert pyruvate to carbon dioxide in the mitochondria for oxidative phosphorylation; under anaerobic conditions, conversion of pyruvate to lactic acid is favoured with relatively low amounts of pyruvate being diverted to the mitochondria. In contrast, cancer cells primarily derive energy from glucose via glycolysis to lactic acid, even under highly aerobic conditions, a property first observed by Otto Warburg 1. This ‘aerobic glycolysis’, also known as the ‘Warburg effect’ 2, is much less energy-efficient than the oxidative phosphorylation pathway 3. It is usually accompanied by marked increases in glucose uptake and consumption 4, a phenomenon commonly exploited in tumour imaging using 18-fluorodeoxyglucose positron electron tomography 5. In addition, cancer cells derive energy from up-regulated non-glucose-dependent pathways, such as increased glutaminolysis under aerobic conditions 2,6,7. Aerobic glycolysis and increased glutaminolysis are collectively regarded as ‘reprogrammed energy metabolism’, a phenomenon now generally accepted as a key metabolic hallmark of cancer 3,8,9. Both pathways lead to the production and secretion of lactic acid, markedly contributing to metabolic acidosis commonly found in solid cancers 2,6,7. Extracellular pH values in tumours can be as low as pH 6.0–6.5, in contrast to pH 7.5 present in normal cell environments 10–12.

## Why do cancers opt for altered energy metabolism?

The preference of cancers for aerobic glycolysis over the more energy-efficient oxidative phosphorylation pathway has been a subject of major interest since it was first observed in the 1920s 13. Many researchers have speculated on the advantages of aerobic glycolysis for cancers, but the causal relationship of this altered metabolism to cancer development is still unclear 3. Otto Warburg speculated that the metabolic alteration was necessitated by a mitochondrial defect in the cancer cells impairing normal oxidative phosphorylation 14. However, further studies have since shown that mitochondrial defects only partially account for the phenomenon. Although certain malignancies indeed harbour mitochondrial defects that make aerobic glycolysis a necessity 15, the majority of cancers are able to revert back to oxidative phosphorylation when lactic acid generation is inhibited 16.

Tumours commonly encounter fluctuating oxygen levels, periodically alternating between normoxic and hypoxic conditions 17. This raises the distinct possibility that aerobic glycolysis has arisen as an adaptation to hypoxic conditions. Use of oxygen-independent glycolysis would confer a proliferative advantage to cancer cells, making them less susceptible to hypoxic stress during episodes of spontaneous hypoxia 13, even if that would come at a cost of energy inefficiency during times of adequate oxygenation. This theory has been extended by suggesting cooperation between normoxic and hypoxic cancer cells within a tumour aimed at maximizing energy efficiency 2. It is proposed that the hypoxic cells are the primary utilizers of glucose, converting it via glycolysis to lactate. Furthermore, lactate secreted by the hypoxic cells would be taken up by normoxic cancer cells and then converted back to pyruvate for oxidation via the citric acid cycle 2,18. This theory, however, does not fully account for the preference of cancers for glycolysis under conditions of abundant oxygenation.

Components of glucose uptake and glycolytic pathways can be up-regulated by oncogenes such as *Ras*, *Akt*, and *Myc* 3. This observation is particularly intriguing, since oncogenic activation is often thought of as an early event in cancer development and progression, and aerobic glycolysis may hence actually predate the onset of hypoxic selection and have a functional role in the early stages of the disease. Other proposals have been put forward, primarily focusing on the mechanisms by which aerobic glycolysis could confer a proliferative advantage to cancer cells 3,13,19. As this pathway is much less efficient than oxidative phosphorylation in generating ATP, ie by approximately 18-fold, the question is raised as to how a reduced supply of ATP can lead to improved proliferative potential. One proposal states that the advantage of aerobic glycolysis lies in incomplete utilization of glucose, allowing upstream intermediates to be redirected for biosynthesis, thereby providing cancer cells with an abundance of building blocks for synthesis of essential cellular components such as macromolecules 19. While such an explanation appears sound, there is still controversy regarding how common such a mechanism is in normal proliferating cells 20. Another proposal states that acidification of the micro-environment by lactic acid, resulting from up-regulation of glycolysis, can be expected to lead to the development of acid-resistant phenotypes exhibiting a powerful, selective growth advantage that promotes unconstrained proliferation and tissue invasion of cancer cells 13.

Increased glutaminolysis would also have several advantages for cancers: glutamine is the most abundant amino acid in plasma and forms an important additional energy source in tumour cells, especially when glycolytic energy production is low; increased availability of the degradation products of glutamine, ie glutamate and aspartate, as precursors for nucleic acid and serine synthesis; and the insensitivity of glutaminolysis to high concentrations of reactive oxygen species 2,6,7.

Of major interest is the finding that the reprogrammed energy metabolism plays an important role in cancer growth-promoting angiogenesis. Angiogenic endothelial cells, like tumour cells, are largely dependent on aerobic glycolysis and increased glutaminolysis for energy. The preference for these pathways allows the development of neovasculature from endothelial, tumour-blood-vessel-lining cells under hypoxic conditions. In addition, lactic acid generated by the pathways has been found to markedly promote angiogenesis by increasing the production of interleukin-8/CXCL8, driving the autocrine stimulation of endothelial cell proliferation and maturation of new blood vessels 21,22.

## Altered energy metabolism and evasion of immune destruction—lactic acid as a critical, regulatory metabolite

Recent studies indicate that altered energy metabolism can also enhance the growth of cancers by promoting tumour evasion of immune destruction. It is now commonly accepted that a transformed, immunogenic cell can only develop into a tumour if it has the ability to evade the cytotoxic immune response that it evokes 8,23. The anti-cancer immune response, as it is mediated by effector T-cells, has long been known to be highly dependent on components of the micro-environment such as helper cells and cytokines. However, it is also influenced by the environmental pH; an acidic pH can markedly impede the function of normal immune cells 24,25. Lowering the environmental pH to 6.0–6.5, as can be found in tumour masses, has been reported to lead to loss of T-cell function of human and murine tumour-infiltrating lymphocytes (eg impairment of cytolytic activity and cytokine secretion); the T-cell function could be completely restored by buffering the pH at physiological values 11.

**Figure 1:**
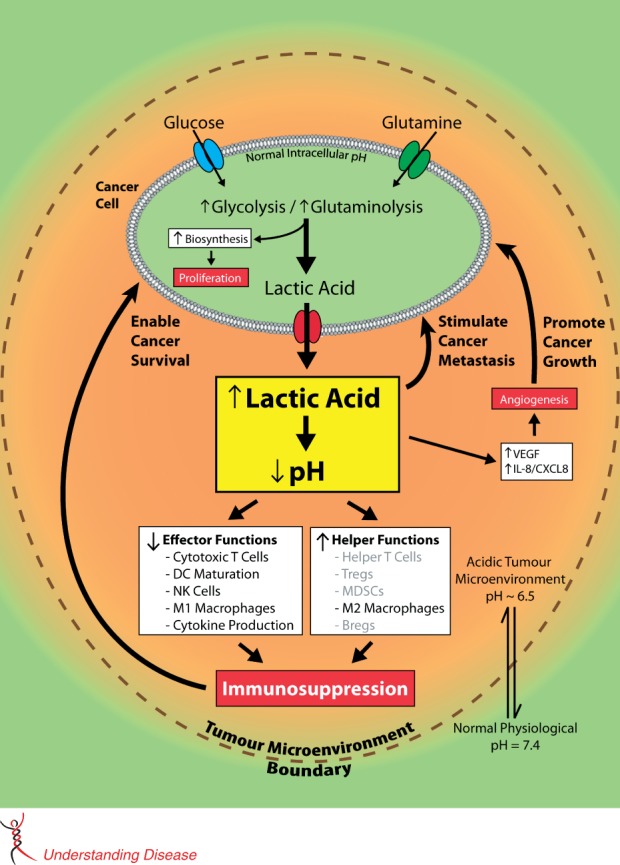
A proposed model for the central, regulatory immunosuppressive role of cancer-generated lactic acid with both experimentally demonstrated (in black) and potential (in grey) consequences. The excess lactic acid produced by cancer cells through reprogrammed metabolism results in an acidified tumour micro-environment. This decrease in pH promotes multiple cancer processes, including angiogenesis, invasion, and metastasis. More importantly, the acidic tumour micro-environment also suppresses the anti-cancer immune response, particularly through decreased cytotoxic T-cell function, reduced dendritic cell maturation, and enhanced helper cell activities. This locally suppressed immunity then enables cancer cells to survive and serves as a basis for subsequent malignant progression. As such, cancer-generated lactic acid promotes tumour evasion of immune destruction and should be viewed as a critical immunosuppressive metabolite rather than a ‘waste product’.

The primary cause responsible for the acidic pH and pH-dependent T-cell function-suppressive effect in a tumour micro-environment has been identified as lactic acid 26–30. It has also been demonstrated that cancer-generated lactic acid and the resultant acidification of the micro-environment increase the expression of ARG1 in tumour-associated macrophages, characteristic of the M2 helper phenotype 31. Furthermore, another study showed that under physiological or slightly alkaline conditions, glycolysis was selectively up-regulated by neuroblastoma cells, whereas oxidative phosphorylation was preferred by the cells when the extracellular pH was acidic; these effects were independent of changes in oxygen concentration or glucose supply 32. Thus, aerobic glycolysis can serve as a negative feedback loop that adjusts the pericellular pH in tumours towards a broad acidic range by increased lactic acid production and secretion. Taken together, the studies suggest that cancer cells can enhance their survival by inhibiting the anti-cancer immune response through actively maintaining a slightly acidic micro-environment. They apparently can do this by altering their energy metabolism to regulate their lactic acid production/secretion 32. The locally suppressed immunity then serves as a basis for the establishment of the malignancy and its subsequent malignant progression (see Figure 1).

In view of the above, researchers are starting to attribute a critical, regulatory role to lactic acid rather than regarding it as a mere metabolic waste product 13,33. Supporting this hypothesis are reports that lactic acid has been found to be a key player in the development and malignant progression of a variety of cancers, with high tumour lactate levels being predictive of metastasis and restricted patient survival 33,34. In addition, lactate dehydrogenase A, the enzyme that catalyses the conversion of pyruvate to lactate, has been found to play an important role in the malignant progression of oesophageal squamous cell carcinoma 35 and tumourigenicity of intestinal-type gastric cancer 36. Furthermore, evidence has been found for involvement of the plasma membrane lactate transporter, MCT4, in breast 37 and prostate 38 cancer.

## Immunosuppressive effect of cancers and epithelial immune cell-like transition (EIT)

As previously reviewed, there apparently are various ways by which epithelial cancers can suppress an anti-cancer immune response 39. We have proposed that their immunosuppressive ability may stem from an acquisition of specific immune cell properties via a transdifferentiation process that we coined ‘epithelial immune cell-like transition’ (EIT) 39. The acquired properties enable cancer cells to suppress the anti-cancer activity of immune cells in their micro-environment by, for example, secreting chemokines to enhance recruitment of T-regulatory cells to the tumour site or producing immunosuppressive cytokines. In this context, the suppression of the immune response by cancers through controlled, lactic acid-induced acidification of their micro-environment appears to be yet another mechanism by which cancers can locally suppress the immune system in an otherwise immunocompetent host.

## Implications for cancer therapy

Aerobic glycolysis and increased glutaminolysis are preferred metabolic energy-generating pathways of cancer 2,8,9 and also have, as mentioned above, an important role in tumour angiogenesis due to the angiogenesis-stimulating effect of lactic acid and the dependence of endothelial cells on this altered metabolism for energy under hypoxic as well as normoxic conditions 21,22. Furthermore, as described above, a growing body of evidence indicates that this altered energy metabolism leads to lactic acid-induced acidification of the cancer micro-environment suppressing the anti-cancer immune response 11,26–30. Targeting these altered metabolic pathways could therefore lead to inhibition of cancer growth based on energy deprivation, in particular under hypoxic conditions, and the growth-inhibitory effects would be especially marked if accompanied by inhibition of tumour angiogenesis and by restoration of the anti-cancer immune response in the micro-environment of the cancers. Additionally, the production of reactive oxygen species (ROS) in the mitochondria is closely tied to normal oxidative metabolism. While the generation of ROS is often associated with tumour progression through damage to DNA and other cellular components, it is suggested that these species play a critical role in regulating various normal physiological processes such as immune function and autophagy 40. As such, the inhibition of lactic acid production in cancer cells may help to re-establish physiological ROS homeostasis and restore normal cellular functions. One drawback of attempting to annihilate cancer through an energy deprivation approach, however, is the likelihood that normal cellular metabolism will also be disrupted; careful pharmacological considerations are therefore required 41. Furthermore, partial inhibition of angiogenesis may lead to normalization of tumour vasculatures, resulting in enhancement of tumour responses to chemotherapy, radiotherapy, and immunotherapy. A window of tumour vascular renormalization would need to be established to maximize the effects of targeting the altered metabolic pathways in combination with other strategies 42,43.

The restoration of the anti-cancer immune response may be accomplished not only through targeting of the two pathways leading to inhibition of lactic acid production 28, but also by interfering with pH regulators such as the proton pump, the sodium–proton exchanger family, and the bicarbonate transporter family 10–12,30,44,45; in this context, it is of interest that regulation of intracellular pH has recently been reported to involve histone acetylation 46. The latter strategies could be useful in combination with immunotherapeutic approaches. Thus far, however, strategies targeting aerobic glycolysis and increased glutaminolysis have often been mainly aimed at inhibiting invasive and metastatic behaviour of malignancies and have not focused on restoring or enhancing the immune response 10,45. Therapeutic effects are commonly examined in experimental immuno-deficient cancer models 47–49, instead of immuno-uncompromised models required to demonstrate effects on the immune response. Key components of the glycolytic pathway have been targeted and such endeavours have been extensively reviewed elsewhere 41,50, with a number of them showing promising results with regard to disease stabilization and tumour regression 49,51.

Altered glucose/glutamine metabolism is only one aspect of tumour metabolism. In addition to lactic acid, other metabolites that suppress T-cell function can be generated by tumours via alterations of lipid and adenosine metabolisms, eg indolamine-2,3-dioxygenase, arginase, inducible nitric oxide synthetase, and lactate dehydrogenase, important players in immune escape mechanisms. Pharmacological blockage of such metabolites could lead to improved cancer therapy by rescuing the endogenous immune response against tumour cells 52,53.

## Conclusions

Lactic acid, commonly generated by cancers via reprogrammed energy metabolism (ie aerobic glycolysis, increased glutaminolysis), has a critical role in their growth as an immunosuppressive metabolite as well as a promoter of angiogenesis. Targeting altered, lactic acid-producing metabolisms of cancers may therefore lead to inhibition of their growth not only via energy/nutrition deprivation, but also through reduction of their immunosuppressive activity in the tumour micro-environment. This strategy, which simultaneously targets two fundamental cancer characteristics (ie altered energy metabolism and evasion of immune destruction), could be effective for the therapy of a broad range of cancers and could be particularly useful in combination with cancer immunotherapy.
